# *Rsad2* is necessary for mouse dendritic cell maturation via the IRF7-mediated signaling pathway

**DOI:** 10.1038/s41419-018-0889-y

**Published:** 2018-08-01

**Authors:** Ji-Su Jang, Jun-Ho Lee, Nam-Chul Jung, So-Yeon Choi, Soo-Yeoun Park, Ji-Young Yoo, Jie-Young Song, Han Geuk Seo, Hyun Soo Lee, Dae-Seog Lim

**Affiliations:** 1 Department of Biotechnology, CHA University, 335 Pangyo-ro, Bundang-gu, Seongnam, Gyeonggi-do 13488 Republic of Korea; 2 Pharos Vaccine Inc., 545 Dunchon-daero, Jungwon-gu, Seongnam, Gyeonggi-do 13215 Republic of Korea; 3 Department of Radiation Cancer Sciences, Korea Institute of Radiological and Medical Sciences, 75 Nowon-ro, Nowon-gu, Seoul, 01812 Republic of Korea; 4 Department of Food Science and Biotechnology of Animal Products, Sanghuh College of Life Sciences, Konkuk University, 120 Neungdong-ro, Gwangjin-gu, Seoul, 05029 Republic of Korea

## Abstract

Dendritic cells (DCs) are the most potent professional antigen presenting cells and inducers of T cell-mediated immunity. However, few specific markers of mature DCs (mDC) have been reported. A previous microarray analysis revealed expression of mDC-specific genes and identified *Rsad2* (radical *S*-adenosyl methionine domain containing 2) as a candidate specific marker for DC maturation. Mouse bone marrow-derived DCs were transfected with *Rsad2* siRNA and examined by flow cytometry, ELISA, western, and confocal microscopy. C57BL/6 mice received intravenously B16F10 cells to establish a pulmonary metastasis model. Tumor-bearing mice then received subcutaneously two injections of mDCs or *Rsad2* knockdown DCs. The cytotoxic T lymphocyte (CTL) population was examined from splenocytes of DC-vaccinated mice by flow cytometry. *Rsad2* was induced at high levels in LPS-stimulated mDCs and mDC function was markedly attenuated under conditions of *Rsad2* knockdown. Moreover, *Rsad2* was necessary for mDC maturation via the IRF7-mediated signaling pathway. The importance of *Rsad2* was confirmed in an *Rsad2* knockdown lung metastasis mouse model in which mDCs lost their antitumor efficacy. Data on the CTL population further supported the results as above. Taken together, *Rsad2* was an obvious and specific marker necessary for DC maturation and these findings will be clearly helpful for further understanding of DC biology.

## Introduction

Dendritic cells (DCs) are the most potent professional antigen presenting cells and have a central role in maintaining immune homeostasis^1^. DCs are not only important for induction of primary immune responses, but may also have a pivotal role in priming of T cell-mediated immune response^2^. Immature DCs (imDCs) capture antigens via pattern-recognition receptors and present them to naive T cells as peptides bound to both major histocompatibility complex (MHC) class I and II molecules; this induces DC maturation and activates T cells. DC maturation cannot be defined simply by measuring a few parameters such as export of MHC class II molecules to the plasma membrane, cytokine secretion, and upregulation of co-stimulatory molecules; this is because all of these can be induced by infectious agents and inflammatory molecules^3^.

Although the powerful antitumor properties of mature (m)DCs have been exploited in several clinical settings, the precise underlying mechanisms and specific markers expressed by these cells remain unclear. In a previous study, we used gene profiling to identify many genes expressed by mDCs^4^. The results suggested that the *Rsad2* (radical S-adenosyl methionine domain containing 2) gene is particularly overexpressed on mDCs rather than other DC subsets; therefore, we investigated its function in DC maturation and DC-mediated immune responses.

*Rsad2* is an interferon-stimulated gene involved in innate immunity and as such is mainly responsible for antiviral responses. It is identical to *cig5* (cytomegalovirus-inducible gene 5) and viperin (virus inhibitory protein, endoplasmic reticulum-associated, interferon-inducible), which have received much attention recently^5^. Some studies have shown that the interferon-stimulated gene (ISG) *viperin* is physically associated with hepatitis C virus (HCV). Moreover, defining the precise mechanism of action of ISG *viperin* that is associated blocking of the HCV replication may present novel therapeutic strategies for HCV^6^. *Rsad2* was first cloned from interferon-treated human macrophages, and is upregulated by viruses, oligonucleotides such as lipopolysaccharide (LPS) and poly I:C, and type I interferons^7–10^. Viperin localizes to the endoplasmic reticulum and lipid droplets (which have an important role in the replication cycle of many viruses), where it inhibits replication of some DNA and RNA viruses^11,12^. TLR3 and TLR4 receptors recognize extracellular dsRNA and LPS, respectively, and induce IRF3-dependent and IRF7-dependent production of type I interferons (IFN-I; IFN-α and -β) on DCs. *Rsad2* also mediates its antiviral function by regulating the TLR7 and TLR9-IRAK1 signaling axis in plasmacytoid DCs^13^.

Although IFN-I are some of the earliest and most potent cytokines released in response to infection, they have an essential role in adaptive immune responses by promoting NK cell or CD8^+^ T cell antiviral cytotoxic activity, either directly or by activating conventional dendritic cells (cDCs)^14–17^. There is some evidence that DC-secreted IFN-I act in an autocrine manner to promote survival of DC precursors and stimulate expression of IFN-I-induced genes in response to pathogen-associated signals^18–20^.

The mechanism underlying *Rsad2*-mediated adaptive immune responses is unclear. *Rsad2* is expressed at high levels in mDCs in response to a wide range of viruses and microbial products such as LPS, suggesting that *Rsad2* is an important component of adaptive T cell responses as well as innate immune responses to diverse pathogens. Thus, *Rsad2* has a strong effect on the ability of mDCs to prime antigen-specific effector T cells. Although our understanding of DC biology is still in its infancy, we are now beginning to use DC-based immunotherapy protocols to elicit immunity responses against cancers and infectious diseases. The aims of this study were to find the role of *Rsad2* in DC biology and rationale for mDC-specific immune regulation.

## Results

### Characterization of bone marrow-derived DCs

A previous report shows that *Rsad2* is required for mDC maturation;^4^ therefore, we first examined immune responses induced by mDCs in vitro. Cultured DCs were generated and divided in two functionally and phenotypically distinct types: immature and mature. First, mouse bone marrow-derived monocytes were cultured with GM-CSF and IL-4 for 8 days to generate imDCs. Incubation of these imDCs for 24 h with LPS (1 µg/ml) led to DC maturation and significant upregulation of cell surface CD40, CD54, CD80, CD86, and MHC class II (Supplementary Fig. 1a). Although imDCs are skillful at endocytosis, they express relatively low levels of surface MHC class II product and co-stimulatory molecules compared with mDCs (e.g., CD86). The mean fluorescence intensity plots show the difference in surface marker expression between imDCs and mDCs (Supplementary Fig. 1a). Cytokine secretion is a mechanism by which DCs regulate immune responses; therefore, we next examined the cytokine profile of DCs. imDCs produced lower levels of pro-inflammatory cytokines (IL-1β, IL-6, TNF-α, and IL-12p70) than mDCs (Supplementary Fig. 1b). Analysis of FITC-conjugated dextran uptake revealed that mDCs showed less phagocytic activity than imDCs (Supplementary Fig. 1c).

We next conducted a series of functional co-culture experiments to investigate the effect of mDCs on T cell proliferation and polarization. To determine whether mDCs promote homeostatic T cell proliferation more efficiently than imDCs, CFSE-labeled naive T cells were co-cultured with purified DC subsets for 72 h. As shown in Supplementary Fig. 1d, mDCs-stimulated T cell proliferation to a greater extent than imDCs. Furthermore, mDCs efficiently induced Th1-mediated immune responses in co-culture experiments (Supplementary Fig. 1e). As previously reported^21^, when co-cultured with mDCs as stimulators, CD4^+^ T cells produce Th1 cytokines (IFN-γ) rather than the Th2 cytokines (IL-4); imDCs were clearly less able to stimulate Th1 cell responses (Supplementary Fig. 1e). Additionally, mDCs were significantly better than imDCs at inducing T cell-mediated production of IFN-γ and IL-17A (Supplementary Fig. 1f). However, no Th2 cytokines (IL-4) were detected. These results indicate that mDCs induce T cell-mediated adaptive immune responses.

### Expression of *Rsad2* in mDCs

**Fig. 1:**
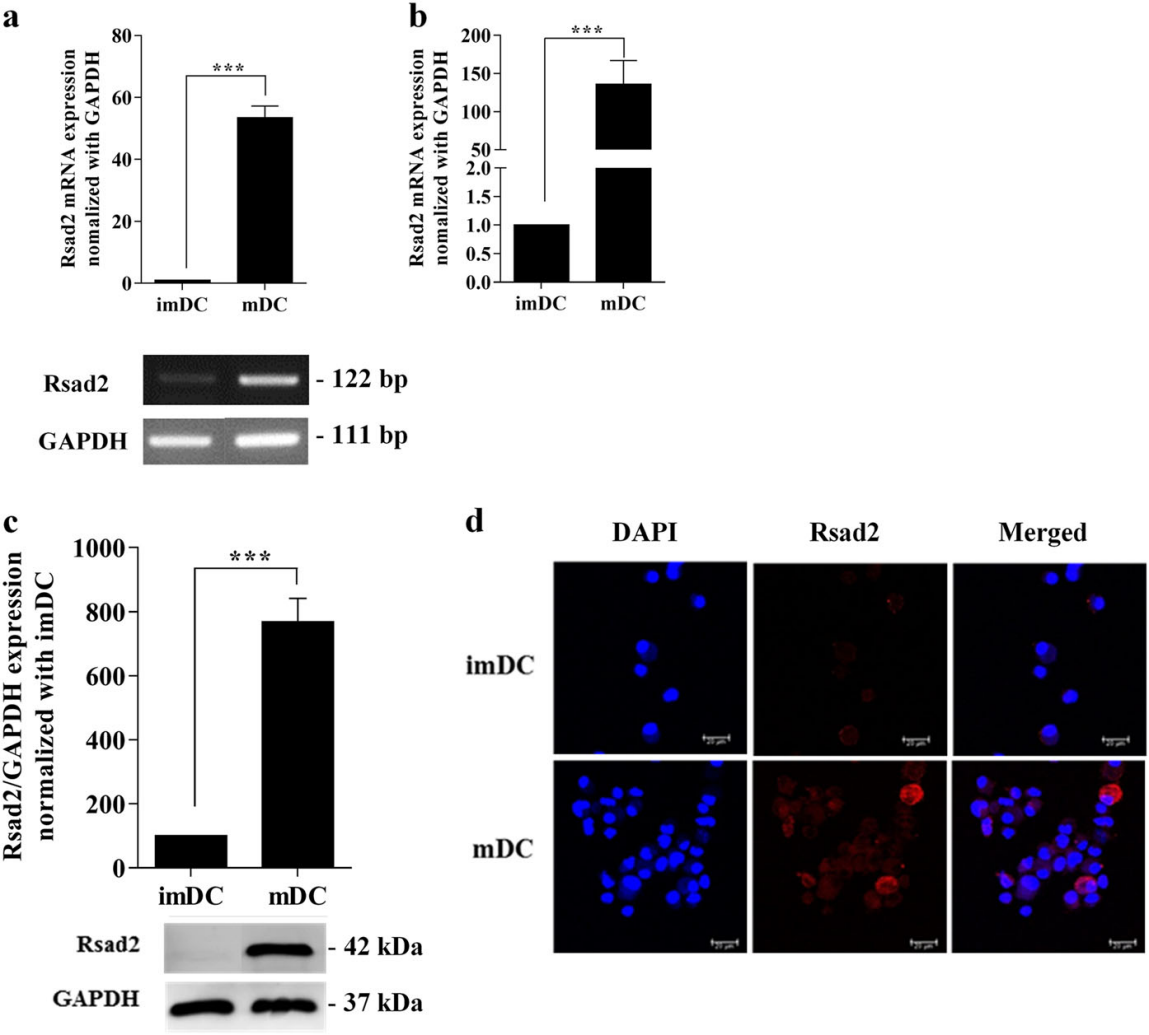
Mature dendritic cells (mDCs) express high levels of *Rsad2*. **a** PCR to detect *Rsad2* expression in DCs. At day 8, DCs were cultured with LPS (1 µg/ml), KLH (10 µg/ml) for mDC, or control medium only for imDC. Values were normalized to *GAPDH* expression. RT-PCR analysis was repeated three times and data expressed as the mean ± SEM. **b** qRT-PCR assay to detect *Rsad2* mRNA expression. The bar graphs show the mean fluorescence intensity, expressed as the mean ± SEM (*n* = 10 independent DC preparations). **c** Western blot analysis of Rsad2 expression. Data are representative of at least three independent experiments. **d** Immunofluorescence analysis of endogenous Rsad2 (red) expression in DCs. Nuclei are stained with DAPI (blue). *P < 0.05, **P < 0.01, and ***P < 0.001, compared with imDC

To determine whether mDCs express *Rsad2*, we cultured mouse bone marrow-derived monocytes with GM-CSF and IL-4 for 8 days to generate imDCs. These cells were treated with LPS to generate mDCs and mRNA was extracted for reverse transcriptase (RT)-PCR and real-time PCR. The results showed that *Rsad2* was expressed by mDCs (Fig. 1a, b, respectively). In addition, western blotting showed that mDCs expressed more Rsad2 protein than imDCs (Fig. 1c). Next, we performed an immunofluorescence assay to examine localization of Rsad2 in mDCs. As shown in Fig. 1d, Rsad2 was detected in the cytoplasmic of mDCs but not in imDCs. These results imply that the *Rsad2* is only endogenously expressed by LPS-stimulated DCs.

### *Rsad2* knockdown mDCs show functional defects

**Fig. 2:**
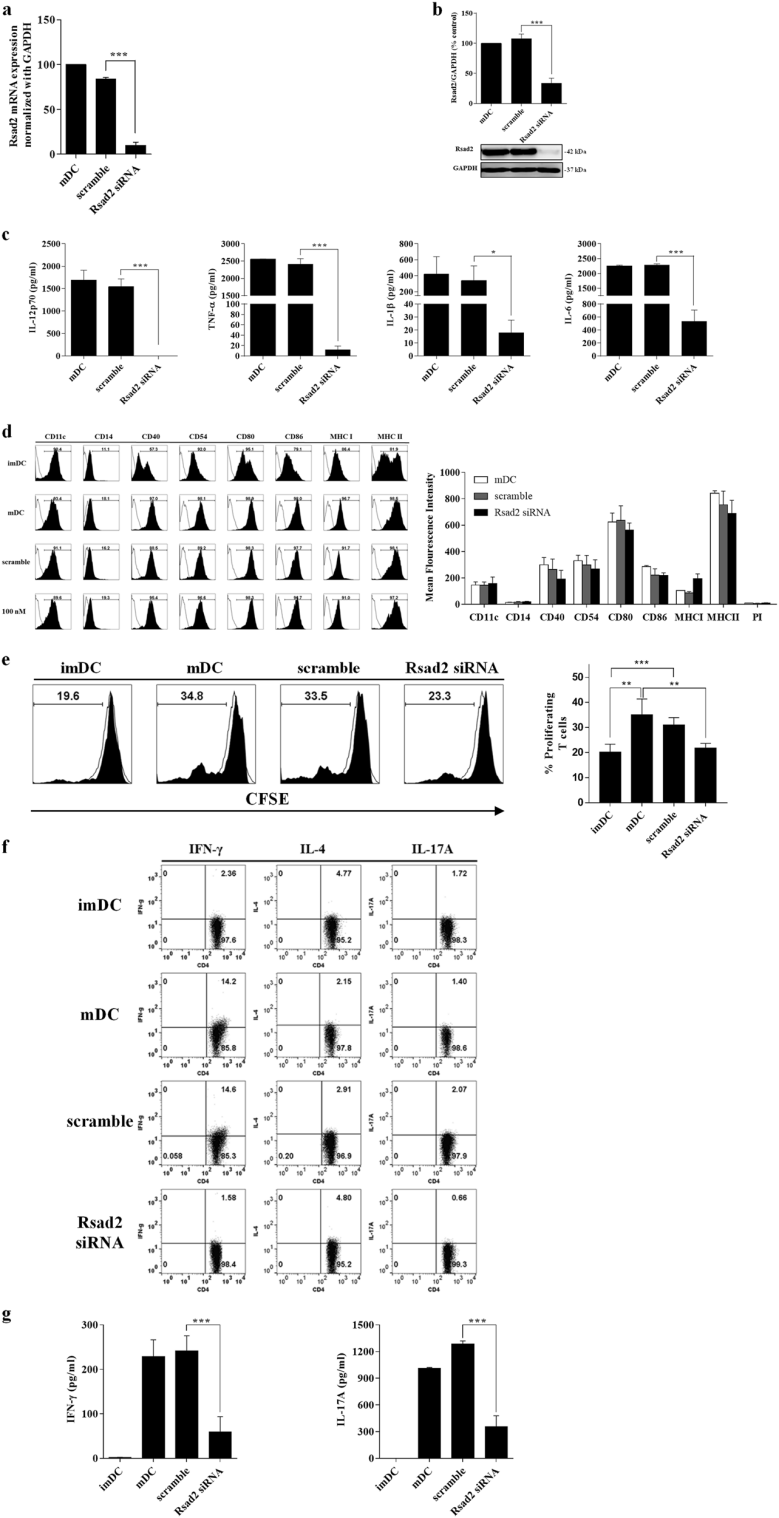
*Rsad2* knockdown mDCs are less efficient at stimulating T cells. **a** qRT-PCR assay to measure *Rsad2* expression in DCs. mDCs were cultured with LPS (1 µg/ml) or KLH (10 µg/ml), or in *Rsad2* or scramble siRNA-transfected. Samples were normalized against *GAPDH* expression. The RT-PCR analysis was repeated three times and data presented as the mean ± SEM. **b** Western blot analysis of Rsad2 knockdown. Data are representative of at least three independent experiments. **c** Cytokine levels in supernatants from DC cultures measured by ELISA. Data are expressed as mean ± SEM (*n* = 10 independent DC preparations). **d** DC subsets (imDCs, mDCs, scramble-mDCs, and *Rsad2* knockdown mDCs) were stained with the fluorescently-conjugated antibodies specific for the indicated molecules and analyzed by flow cytometry. Data are presented as histograms (data are representative of ten independent DC preparations). The bar graphs show the mean fluorescence intensity, expressed as the mean ± SEM (*n* = 10 independent DC preparations). **e** Each DC subset was co-cultured with CFSE-labeled CD3^+^ T cells and T cell proliferation measured after 72 h. The stimulator:responder ratio was 1:10. The bar graphs show the mean fluorescence intensity, expressed as the mean ± SEM (*n* = 10 independent DC preparations). **f** T cell subpopulations were analyzed by flow cytometry. DCs were co-cultured with CD3^+^ T cells and the Th1 (CD4^+^IFN-γ^+^), Th2 (CD4^+^IL-4^+^), and Th17 (CD4^+^IL-17A^+^) cell population were detected by flow cytometry. **g** Measurement of Th1/Th17 cytokines in culture supernatants from DCs/T cell co-cultures by ELISA. Data are expressed as mean ± SEM (*n* = 10 independent DC preparations). *P < 0.05; **P < 0.01; ***P < 0.001, compared with imDC

To confirm whether the *Rsad2* knockdown affects the function of mDCs, we transfected cells with *Rsad2* siRNA (100 nM for 48 h) or scramble siRNA. Our preliminary studies showed that transfecting *Rsad2-*specific siRNA into DCs yielded the greatest protein knockdown at 48 h. The efficiency of siRNA-mediated *Rsad2* knockdown was confirmed by qPCR and western blotting (Fig. 2a, b). Although the DCs had been stimulated with LPS for 24 h, knockdown of *Rsad2* led to a significant reduction in their secretion of pro-inflammatory cytokines (Fig. 2c). However, similar levels of cell surface marker expression were detected on mDCs and *Rsad2* knockdown mDCs. Indeed, *Rsad2* knockdown mDCs and mDCs showed similar expression of CD80 and CD86, as well as MHC class II (or slightly higher expression in mDCs) (Fig. 2d).

Next, to clarify whether *Rsad2* knockdown mDCs lose the ability to stimulate T cells, we co-cultured *Rsad2* knockdown cells with T cells. As shown in Fig. 2e, T cell proliferation in the presence of DCs transfected with *Rsad2* siRNA was markedly reduced at 72 h. In addition, the Th1 T cell population was markedly depleted at 72 h (Fig. 2f). Parallel assays revealed that T cell-mediated secretion of IFN-γ was also reduced by 80% at 72 h (a similar propensity in the IL-17A secretion) (Fig. 2g). Taken together, these results clearly demonstrate that *Rsad2* has a marked effect on the interaction between mDCs and T cells.

### *Rsad2* is required for IRF7-mediated type I IFNs secretion by mDCs

**Fig. 3:**
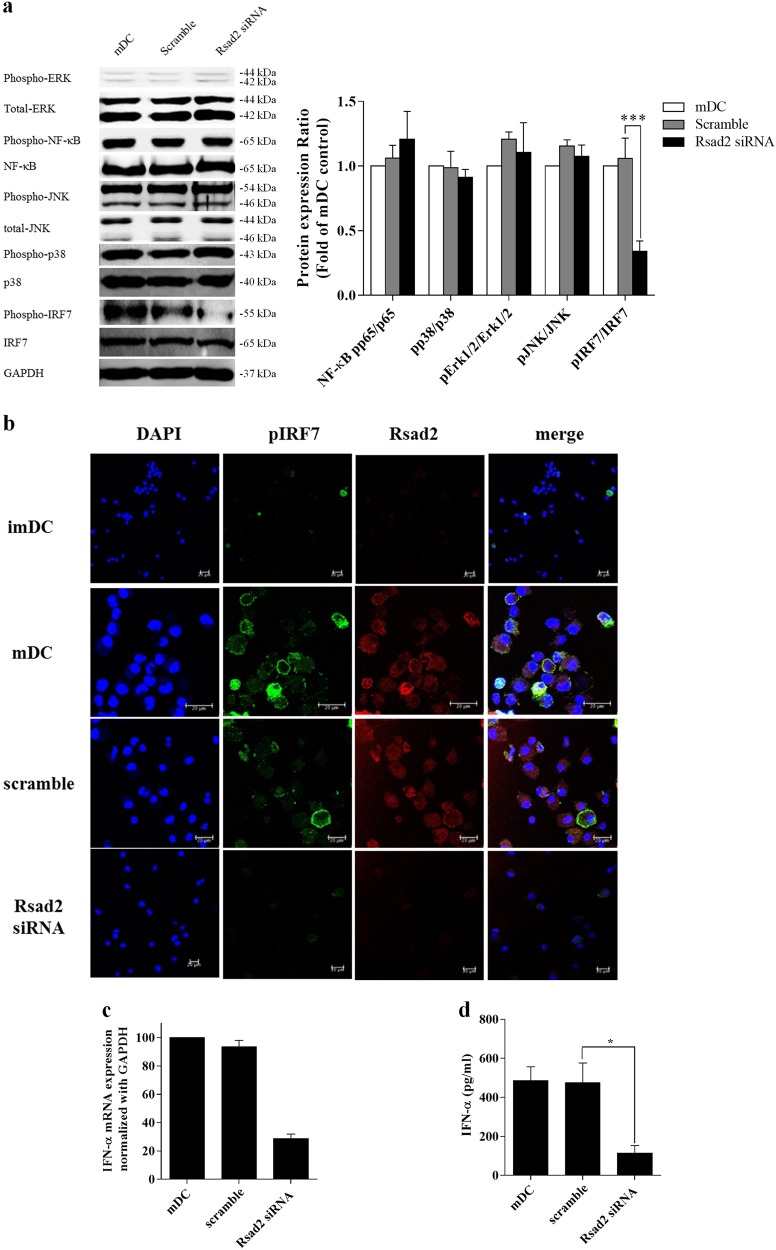
*Rsad2* is required for IRF7-mediated secretion of type I IFN by mDCs. **a** Western blot analysis of MAPKs, NF-κBp65, and IRF7. Data are representative of at least three independent experiments. **b** Immunofluorescence analysis of endogenous Rsad2 (red) and p-IRF7 (green) expression in DCs. Nuclei are stained with DAPI (blue). **c** qRT-PCR assay measuring type I IFN expression by each DC subset. Sample data were normalized against GAPDH expression. RT-PCR analysis was repeated three times and data presented as the mean ± SEM. **d** IFN-α (an IFN-I cytokine) levels in the supernatant of each DC subset measured by ELISA. Data are expressed as mean ± SEM (*n* = 10 independent DC preparations). *P < 0.05, **P < 0.01, and ***P < 0.001

As shown above, knockdown of *Rsad2* abrogated mDC-mediated immune responses. Therefore, we conducted western blot analysis to identify the signaling molecules involved. We examined phosphorylation of NF-κB, ERK, JNK, p38, and IRF7 that are essential in regulating many cellular processes including inflammation, cell stress response, cell differentiation, cell division, cell proliferation, metabolism, motility, and apoptosis. The basal levels of NF-κB and MAPK in freshly isolated mDCs and *Rsad2* knockdown mDCs were similar (Fig. 3a). However, *Rsad2* knockdown mDCs showed a marked defect in expression of phosphorylated IRF7 (Fig. 3a). Therefore, we conducted immunofluorescence analysis to visually show an association with IRF7 in mDCs. As shown in Fig. 3b, mDCs expressed Rsad2; importantly, reduced phospho-IRF7 expression was observed in *Rsad2* knockdown mDCs. These results indicate that Rsad2 contribute to a process of DC maturation via the IRF7-mediated signaling pathway.

Given that *Rsad2*-mediated expression of IRF-3 and IRF-7 in macrophages contributes to IFN-I production by these cells^22^, we suspected that deficient *Rsad2* expression in mDCs would have an effect on IFN-I secretion. Indeed, we found reduced amounts of *IFN-I* mRNA in *Rsad2* knockdown mDCs (Fig. 3c). Consistent with this, mDCs secreted significantly more IFN-I cytokine than *Rsad2* knockdown mDCs (Fig. 3d).

### *Rsad2*-mediated induction of CTL responses

**Fig. 4:**
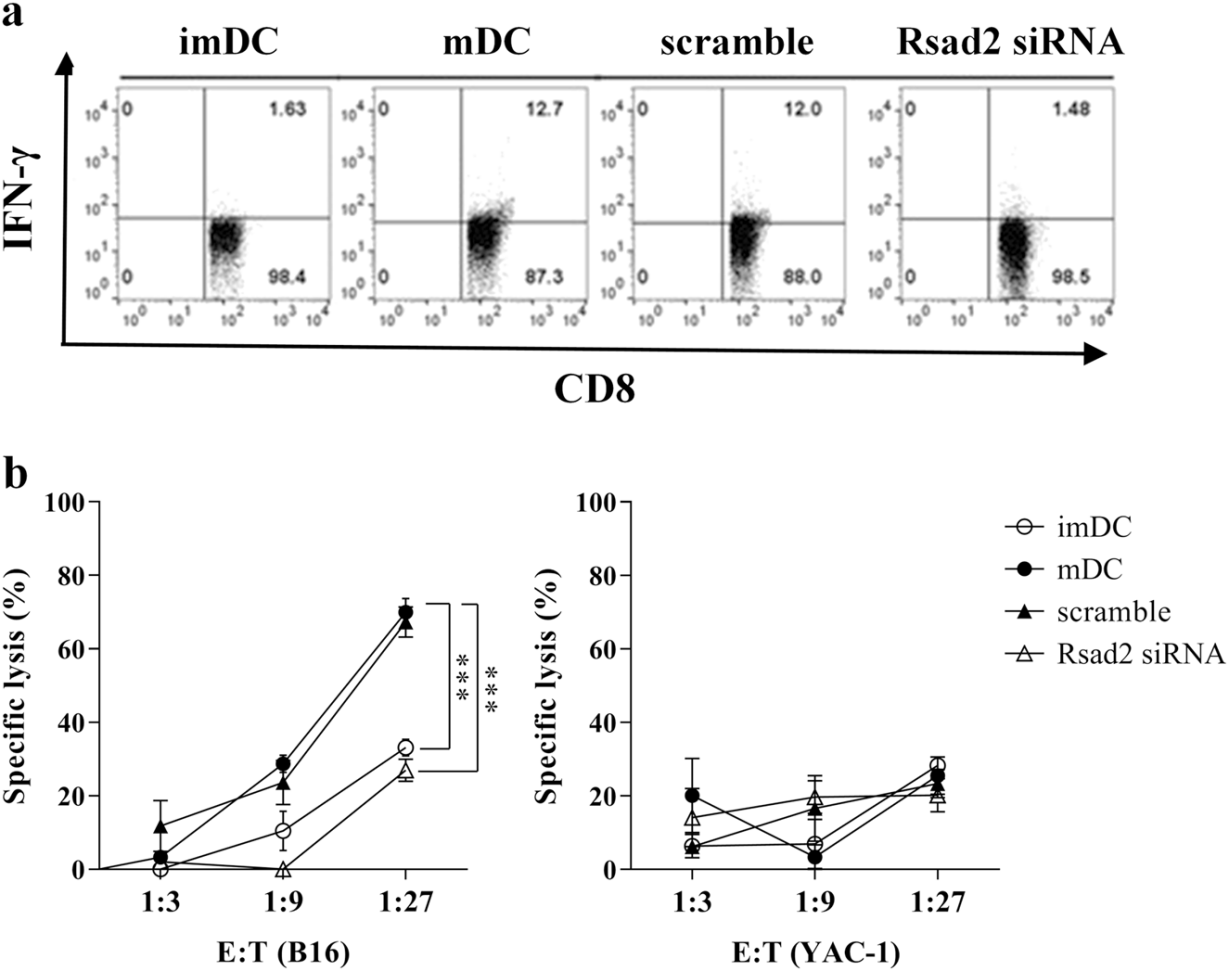
Rsad2-mediated induction of CTL responses. **a** Each DC subset was co-cultured with CD3^+^ T cells for 14 days in the presence of IL-2 (100 U/ml). The stimulator:responder ratio was 1:10. The B16F10-specific IFN-γ^+^CD8^+^ T cell population was then analyzed by flow cytometry. **b** LDH assay to measure primary B16F10-specific CTL responses to each DC subset. The line graphs show the mean fluorescence intensity, expressed as the mean ± SEM (*n* = 5 independent preparations)

We next determined whether loss of *Rsad2* affects induction of CTL and their cytotoxicity. The percentage of CD8^+^IFNγ^+^ T lymphocytes was determined by flow cytometry. As shown in Fig. 4a, T cells co-cultured with B16F10 cell lysate-pulsed mDCs contained a higher percentage of CD8^+^IFN-γ^+^ T lymphocytes than those co-cultured with B16F10 cell lysate-pulsed *Rsad2* knockdown mDCs.

Next, we measured release of cytosolic LDH into the culture medium by a damaged B16F10 melanoma cell line to examine the cytotoxic activity of T cells co-cultured with B16-specific mDCs. B16F10 lysate-pulsed mDC-stimulated T cells (effector cells) were co-cultured with B16F10 melanoma cells (target cells) at effector-to-target cell ratios of 3:1, 9:1, and 27:1. As shown in Fig. 4b, B16F10 lysate-pulsed mDC-stimulated T cells induced greater cytotoxic responses than B16F10 lysate-pulsed *Rsad2* knockdown mDCs-stimulated T cells. However, co-culture with another cancer cell line (YAC-1) did not affect CTL cytotoxicity. Neither B16F10 melanoma cells nor naive T cells cultured alone in control medium (negative controls) released any LDH at 4 h. These results suggest that high-activity CTLs were induced by co-culture with B16F10-pulsed mDCs but not by co-culture with *Rsad2* knockdown cells. To this end, key concepts of *Rsad2*-mediated phosphorylation of IRF7 in the mDCs, resulting antigen-specific T cell induction.

### B16F10 lysate-pulsed mDCs show stronger antitumor effects in vivo than lysate-pulsed *Rsad2* knockdown mDCs

**Fig. 5:**
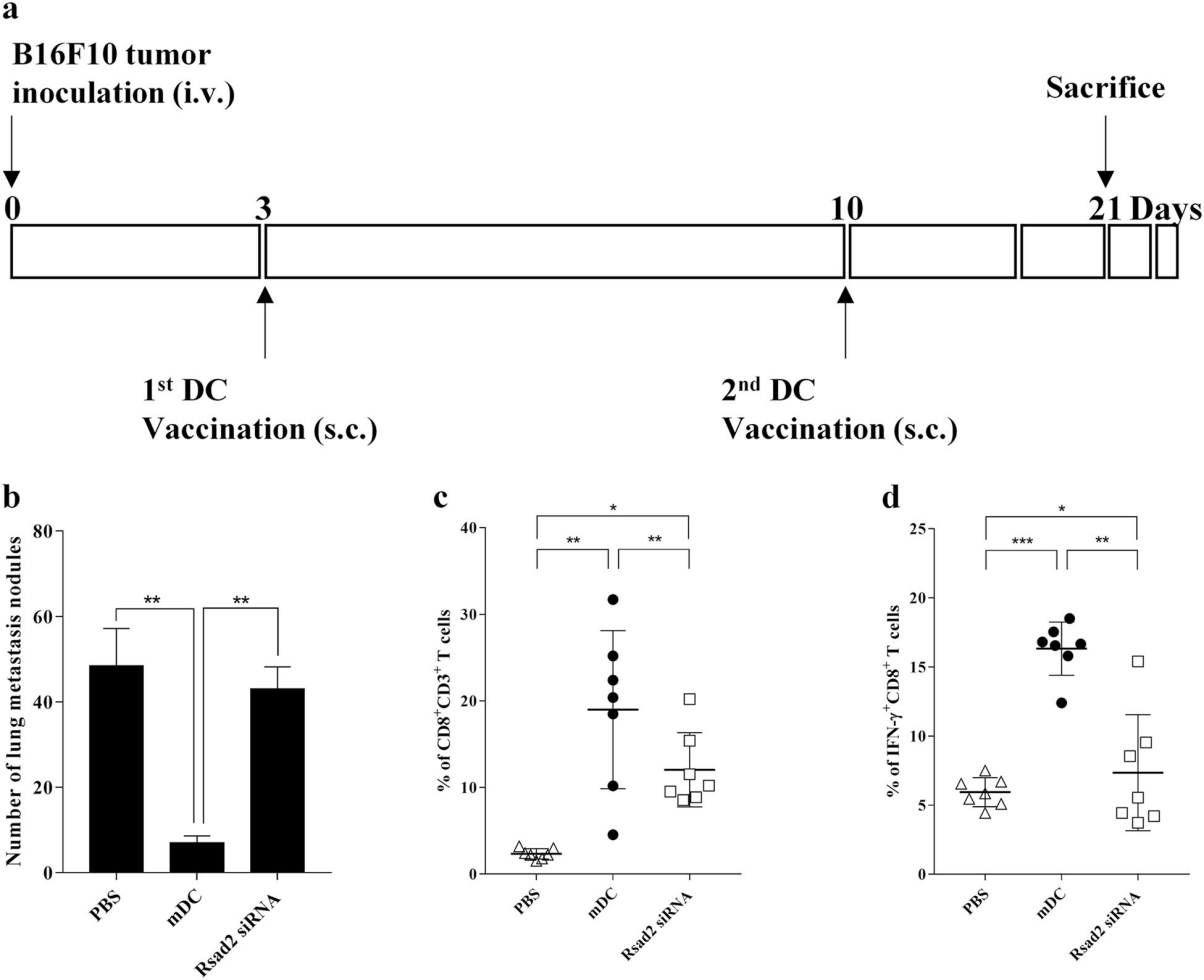
*Rsad2* knockdown mDCs show reduced antitumor activity in a murine model of lung metastasis. Inhibition of pulmonary metastasis by vaccination with B16F10 tumor lysate-pulsed DCs. C57BL/6 mice received viable B16F10 cells (1 × 10^6^) via the lateral tail vein to establish a pulmonary metastasis model. Mice were then inoculated twice intravenously with B16F10 tumor lysate-pulsed DCs (1 × 10^6^) (once on day 3 and once on day 10 post-tumor cell tumor injection). Mice were killed on day 21 **a** DC vaccination schedule. **b** Tumor nodules were counted 21 days after killing tumor-bearing mice. Data represent the mean number of nodules ± SEM (*n* = 10 mice per group). **c** Splenocytes from DC-vaccinated mice were stained with the anti-CD8 and anti-CD3 antibody. The graph show the number of CD8^+^/CD3^+^ T cells in each mice group, expressed as the mean ± SEM (*n* = 7 per each group). **d** Splenocytes from DC-vaccinated mice were stained with a FITC-anti-CD8 antibody, fixed with 2% formaldehyde, permeabilized with 0.5% saponin, and incubated with PE-anti-IFN-γ. The graph shows the percentage of the IFN-γ^+^CD8^+^ T cells in each mice group, expressed as the mean ± SEM (*n* = 7 per each group). *P < 0.05, **P < 0.01, and ***P < 0.001

Finally, to examine antitumor responses in vivo, B16F10 tumor-bearing mice were immunized with mDCs or *Rsad2* knockdown mDCs and survival evaluated. Mice bearing B16F10 tumors were vaccinated with mDCs on days 3 and 10 after initial tumor cell injection (Fig. 5a). As shown in Fig. 5b, mice treated with B16F10-pulsed mDCs showed little or no growth of B16F10 tumors in the lungs; by contrast, mice treated with PBS alone or with *Rsad2* knockdown mDCs harbored many B16F10 tumors.

Next, to compare differences in the in vivo immune responses in mice vaccinated with B16F10-pulsed mDCs or *Rsad2* knockdown mDCs, we analyzed IFN-γ-producing CD8 T cell populations. Figure 5c shows that vaccination with B16F10-pulsed *Rsad2* knockdown mDCs induced more reduced CD8 T cell populations than lysate-pulsed mDCs. Moreover, vaccination with B16F10-pulsed mDCs resulted in potent stimulation of IFN-γ-secreting CD8^+^ T cells (Fig. 5d). The results revealed that i.v. immunization with B16F10 cell lysate-pulsed mDCs protected mice against formation of B16F10 melanomas. These results imply that *Rsad2*-expressing mDCs induce a potent antitumor response.

## Discussion

One of the most critical features of DC biology is functional maturation, which influences the fundamental properties of an immune response^1^. Two major types of DC are present under inflammatory conditions: imDCs and mDCs. These DC subsets express different levels/types of pro-inflammatory cytokines and show differing abilities to stimulate T cell proliferation and IFN-γ-mediated CTL responses^3^. Although these different phenotypes provide clues as to cellular function, the different DC subsets share some features under inflammatory conditions. Therefore, there is a need to identify reliable DC markers that will enable identification of different DC subsets^21,23^.

Among innate inflammatory cytokines, IFN-I are considered major players that link innate and adaptive immunity by activating DCs. IFN-I promote expression of co-stimulatory molecules, and IFN-I-treated DCs prime T cells more effectively in vitro^17,24–26^. *Rsad2* has a dual function: direct suppression of viral replication and facilitation of TLR7-mediated and TLR9-mediated production of IFN-I^27^. A study based on human monocyte-derived DCs found that *Rsad2* is expressed at higher levels in type 2 DCs, which have the ability to induce differentiation of naive T cells into Th2 cells by activating the NF-κB and AP-1 signaling pathways^28^. Additionally, *Rsad2* shows direct and indirect antiviral activity against various viruses and is induced directly in myeloid DCs by IRF-3-mediated and 7-mediated production of type 1 IFN^11,12,22^.

Here, we show that expression of *Rsad2*, a typical interferon-stimulated gene (ISG), was upregulated in fully matured DCs. To understand how *Rsad2* induces mDC function, we generated *Rsad2* knockdown cells. *Rsad2* knockdown mDCs lost the ability to promote pro-inflammatory cytokine production and induce T cell proliferation. Next, we confirmed that phosphorylation of IRF7 has a role in *Rsad2*-mediated mDC function. Overall, the results provide insight into the role of *Rsad2* in mDC function and IRF7-mediated production of IFN-I. Thus, *Rsad2* is not only involved in antiviral innate immune responses, but is also a powerful stimulator of adaptive immune response mediated via mDCs. Furthermore, it is required to confirm reproducibility of these results by using human DCs. *Rsad2* is a specific marker for DC maturation, overexpression of which may lead to development of powerful cell-based immunotherapies.

## Materials and methods

### Ethical approval

All protocols involving the use of animals were approved by the Institutional Animal Care and Use Committee of CHA University (Project No. IACUC160095), and all experiments were carried out in accordance with these approved protocols.

### Mice

Male C57BL/6 mice (6–8 weeks-of-age, each weighing 14–16 g) were purchased from Orient Bio Inc. (Gyeonggi, Republic of Korea). All mice were housed in a temperature- and humidity-controlled room under a 12 h/12 h light/dark cycle.

### Generation of DCs

DCs were generated from bone marrow progenitors obtained from 6-week-old C57BL/6 mice, as described previously^4^. Briefly, bone marrow single cell suspensions were prepared as described^4^, washed with PBS (Lonza, Basel, Switzerland), and cultured in RPMI 1640 (Lonza) supplemented with 10% FBS (Corning, NY, USA), 1 × antibiotics (Gibco, Life Technologies, Grand Island, NY, USA), 20 ng/ml recombinant mouse (rm) GM-CSF (JWCreaGene, Gyeonggi, Korea) and 2 ng/ml rmIL-4 (JWCreaGene). Cells were cultured at 37 °C under 5% CO_2_. After 3 days, another 10 ml of complete medium containing GM-CSF was added to each dish. On day 6, 50% of the culture medium was replaced with fresh medium containing the same concentrations of GM-CSF and IL-4. On day 8, the non-adherent cells were harvested and mDCs treated for 24 h with 1 µg/ml LPS (Sigma-Aldrich, St Louis, MO, USA) or 10 µg/ml Keyhole Limpet Hemocyanin (KLH) (Sigma-Aldrich). For the lactate dehydrogenase (LDH) release assays and in vivo studies, tumor cell lysates were used as a source of antigen.

### Flow cytometry analysis

Phenotypic analysis was performed by direct immunofluorescence staining of DC cell surface markers. Cells (in PBS) were stained with the following antibodies at 4 °C for 20 min: Phycoerythrin (PE)-conjugated anti-CD11c (Clone N418); Fluorescein isothiocyanate (FITC)-conjugated anti-CD14 (Clone Sa14-2); PE-conjugated anti-CD40 (Clone 3/23); FITC-conjugated anti-CD54 (Clone 3E2); PE-conjugated anti-CD80 (Clone 16-10A1); FITC-conjugated anti-CD86 (Clone PO3); FITC-conjugated anti-MHC I (Clone KH95), or PE-conjugated anti-MHC II (Clone 2G9) (all antibodies were purchased from BD Pharmingen, San Diego, CA, USA), plus appropriate isotype controls. Cells were then analyzed using a 3-color FACSCalibur cytometer (BD Biosciences, Mountain View, CA). Data were collected from 10,000 events and analyzed using FlowJo10 software (BD Biosciences).

### Cytokine measurement

The concentrations of interleukin (IL)-1β, IL-6, IL-10, IL-12p70, and IL-4 secreted by lymphocytes or DCs were measured using commercially available ELISA kits (all kits were purchase from BioLegend, CA, USA) in accordance with the manufacturer’s instructions.

### Dextran uptake assay

Phagocytic activity was examined as previously described^29^. Briefly, immature DCs and mDCs were incubated with FITC-conjugated dextran (1 mg/ml; Sigma-Aldrich) in culture medium for 1 h at 37 °C. Next, cells were washed with PBS prior to analysis by flow cytometry (FACSCalibur™; BD Biosciences). Cells positive for FITC (detected by fluorescence detector 1) were identified as cells that had engulfed dextran.

### Quantitative real-time PCR (qRT-PCR)

RNA was isolated using LABOzol, according to the manufacturer’s instructions (Invitrogen, California, USA). RNA (1 µg) was then used to synthesize cDNA using a LaboPass cDNA synthesis kit (Cosmogenetech, Seoul, Korea). cDNA samples were subjected to real-time PCR analyses with *Rsad2* primers and a SensiFAST SYBR Hi-ROX-kit (Bioline, London, UK) using the real-time quantitative RT-PCR (Applied Biosystems, Foster City, CA, USA). The primer sequences were as follow: *Rsad2* forward, 5′-GGTGCCTGAATCTAACCAGAAG-3′ and *Rsad2* reverse, 5′-CCACGCCAACATCCAGAATA-3′; *GAPDH* forward, 5′-AACAGCAACTCCCACTCTTC-3′ and reverse, 5′-CCTGTTGCTGTAGCCGYATT-3′. The normalized value for *Rsad2* mRNA expression were calculated as the relative quantity of *Rsad2* divided by the relative quantity of *GAPDH*. All samples were run in triplicate.

### Western blot analysis

Whole cell lysates for western blot were prepared as described^30^. Protein concentrations of whole cell lysates were measured using a Bradford assay kit (Sigma-Aldrich). Equal amounts of protein were loaded into the wells of a SDS-PAGE gel and the separated proteins transferred to PVDF membranes (Thermo Scientific, Hudson, NH, USA). The membranes were blocked with 10% (w/v) skim milk in PBST and then incubated with primary antibodies (anti-Rsad2 (Abcam, Cambridge, UK), p-Erk1/2, Erk1/2, p-NF-κBp65, NF-κBp65, p-JNK, JNK, p-p38, p38, p-IRF7, IRF7, and GAPDH; all diluted 1:1000; Cell Signaling Technologies, Danvers, MA, USA) overnight at 4 °C. The membranes were then washed with PBST and incubated with an HRP-conjugated rabbit anti-mouse IgG and mouse anti-goat secondary antibody (diluted 1:2000; Cell Signaling Technologies) for 2 h at room temperature. The membrane was then exposed to ECL reagents (Thermo Scientific) and the resulting signals detected using a Luminescent image analyzer (LAS-4000; Fuji Film, Tokyo, Japan).

### RNA interference

For delivery of small interfering RNAs (*Rsad2* siRNAs; sense: 5′-GCAGAAAGAUUUCUUAUAA-3′; antisense: 5′-UUAUAAGAAAUCUUUCUGC-3′), DCs were plated into each well of a 6-well plate (SPL Life Sciences, Gyeonggi, Republic of Korea) and transfected with siRNA duplexes using the Lipofectamine 3000 transfection reagent (Invitrogen), according to the manufacturer’s instructions. Briefly, cells were cultured for 4 h with the transfection mixture in OptiMEM medium (Invitrogen). Next, a volume of medium equal to that of the Lipofectamine-100 nM *Rsad2* siRNA mixture was added. Four hours later, the cells were plated into 24-well dishes and stimulated with 1 μg/ml LPS. The next day, the cells were subjected to a second round of transfection to increase siRNA-mediated gene suppression. At 24 h post-second transfection, the cultures were harvested and prepared for further analysis.

### CFSE proliferation assay

Splenocytes were isolated from wild type C57BL/6 mice spleen and disaggregated in RPMI 1640 medium. CD3^+^ T cells were isolated by passing the splenocytes through a nylon wool (Polysciences Inc., Warrington, PA, USA) column. Nylon-wool column-purified T cells from spleens were labeled using CFSE labeling kits (Molecular Probes, Oregon, USA) according to the manufacturer’s instructions. Cells were then co-cultured with DCs at a DC:T cell ratio of 1:10. CFSE-labeled purified CD3^+^ T cells (1 × 10^6^ cells/ml) were used as responders and imDCs or mDCs (1 × 10^6^ cells/ml) were used as stimulators. Cells were co-cultured at 37 °C for 72 h in 2 ml of RPMI 1640 supplemented with 10% FBS.

### Generation of CTLs

DCs loaded with B16F10 cell lysates were sorted and used as stimulatory cells, while CD3^+^ T cells in a final volume of 1 ml. The RPMI 1640 media was supplemented with 10% FBS and IL-2 (10 IU/ml at weeks 2 and 3 of culture). T cells were re-stimulated weekly (for an additional 2 weeks) with B16F10 lysates loaded or unloaded DCs. Six days after the last stimulation, T cells were harvested and cytotoxic activity and the capacity for IFN-γ release tested.

### Preparation of tumor cell lysates

Confluent cultures of B16F10 or YAC-1 cells were incubated for 10 min with 0.25% trypsin-EDTA solution (Gibco, Life Technologies), carefully detached, washed twice in PBS, and resuspended at a density of 5 × 10^6^/ml in serum-free DMEM (Lonza). The cell suspensions were frozen at −80 °C, and lysed by repeated (four times) freeze-thaw cycles. To remove crude debris, the lysate was centrifuged for 10 min at 300×*g*. The supernatant was then collected and passed through a 0.2-μm filter (Sigma-Aldrich). The protein concentration of the lysate was measured in a commercial assay (Bio-Rad, Munich, Germany).

### Cytotoxicity assay

The CytoToxw96 Non-Radioactive Assay kit (Promega, WI, USA) was used to measure T cell-mediated cytotoxicity. CTLs (effector cells) and B16F10 tumor cells (target cells) were co-cultured in round bottom 96-well plates for 4 h in 37 °C/5% CO_2_. Next, lysis solution (20 μl; 10 ml/100 ml) was added to four wells and incubated for 45 min. These wells acted as positive controls (maximum release of LDH). Next, 50 µl of supernatant from each well was placed in a new 96-well plate and 50 µl of reconstituted substrate mix added. The plates were then kept in a dark room at room temperature for 30 min. Stop solution (50 µl) was then added to each well for 1 h. Absorbance (490 nm) was recorded using a Dynatech MR 4000 plate reader (Dyantech laboratories Billingshurst, UK). Each experiment was repeated six times. Cytotoxicity was evaluated with the CytoTox96 Non-Radioactive Cytotoxicity Assay kit (Promega) following the manufacturer’s protocol; the colorimetric assay quantifies lactate dehydrogenase (LDH) activity released from the cytosol of damaged target cells into the supernatants.

### Confocal microscopy analysis

Confocal analysis was conducted as previously described^24^. After fixation for 10 min in 4% paraformaldehyde in PBS, cells were washed in 1% Tween20/PBS and permeabilized with 0.1% Triton-X 100 (Sigma-Aldrich) in PBS. Cells were then incubated in 5% NGS in room temperature for blocking. After washing with 1% Tween20 in PBS, cells were incubated for 24 h at 4 °C with an anti-Rsad2 antibody (Abcam). After further washing in 1% Tween20 in PBS, cells were stained for 2 h at room temperature with a secondary antibody (Alexa fluor 488 or 555-conjugated anti-mouse Ab (DAKO, Santa Clara, CA, USA)) in DAKO antibody diluent solution (DAKO). Cells were then resuspended in DAPI (DAKO). Confocal microscopy was performed using a Leica TCS-NT SP equipped with argon, krypton, and helium/neon lasers, and a spectrophotometer was used to separate the detection channels of Alexa fluor488 (450–500 nm) and Alexa fluor555 (580–660 nm).

### Immunization of mice and tumor challenge

B16F10 cells were trypsinized, washed twice in 1 × PBS to eliminate serum proteins, and suspended in PBS (at 1 × 10^7^ cells/ml) prior to injection. Male C57BL/6 mice (7–10 weeks old) were challenged intravenously (i.v.) with 100 µl (1 × 10^6^ cells) of B16F10 cell. After 3 days, B16F10 lysate (~100 µg of proteins)-pulsed mDCs (1 × 10^6^ cells) were injected into the subcutaneously (s.c.) once on day 3 and once on day 10. Mice were killed on day 21. The lungs were removed and washed for 10 min in Bouin’s fluid (Sigma-Aldrich). The lobes of the lungs were separated and the total number of superficially visible tumor cell colonies per lung was counted. Lungs containing tumor nodules were photographed.

### Statistical analysis

All assays were performed in triplicate (at least). Data are expressed as the mean ± standard error of the mean (SEM). Data were analyzed by one-way ANOVA and a *P* value < 0.05 was considered statistically significant. All statistical analyses were performed using Prism software (GraphPad Prism v5.0; GraphPad Prism Software, San Diego, CA).

## Electronic supplementary material

Supplemental Figure-Characterization of bone marrow-derived dendritic cell (DCs)

Supplementary figure legends
