# Supplementary figures and images for: Rsad2 is necessary for mouse dendritic cell maturation via the IRF7-mediated signaling pathway
Source: Cell Death Dis. 2018 Aug 1;9(8):823. doi: 10.1038/s41419-018-0889-y (PMC6070531; doi:10.1038/s41419-018-0889-y)

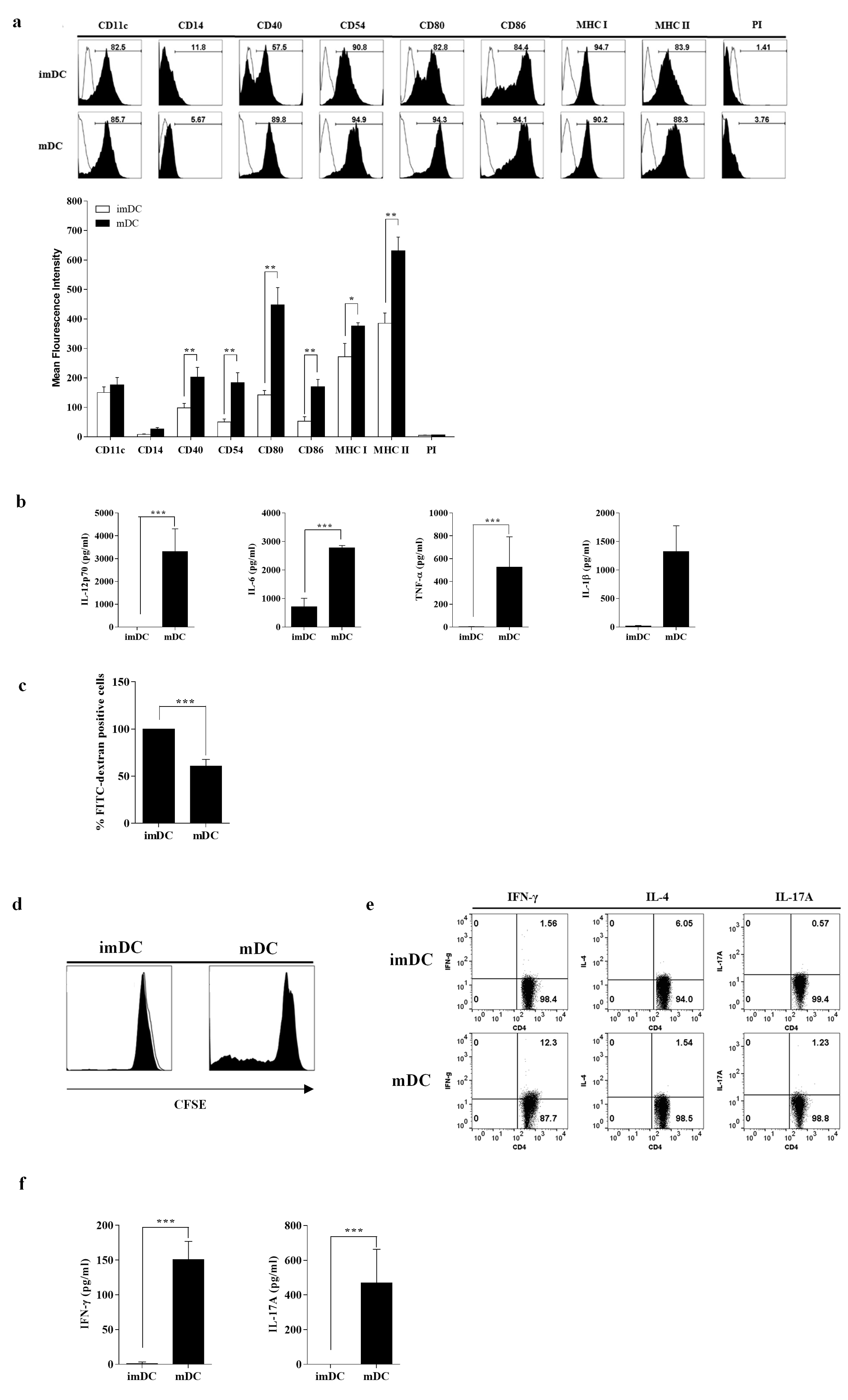

Supplement: Supplementary file 1 — Supplemental Figure-Characterization of bone marrow-derived dendritic cell (DCs) [file 41419_2018_889_MOESM1_ESM.tif]
