# Supplementary material for: Rsad2 is necessary for mouse dendritic cell maturation via the IRF7-mediated signaling pathway
Source: Cell Death Dis. 2018 Aug 1;9(8):823. doi: 10.1038/s41419-018-0889-y (PMC6070531; doi:10.1038/s41419-018-0889-y)
Supplement: Supplementary file 2 — Supplementary figure legends [file 41419_2018_889_MOESM2_ESM.docx]

**Supplementary Fig. 1** Characterization of bone marrow-derived dendritic cells (DCs). (a) DC subsets (imDCs and mDCs) were stained with fluorescently-conjugated antibodies specific for the indicated molecules and analyzed by flow cytometry. Data are presented as histograms (representative of ten independent DC preparations). The bar graphs show the mean fluorescence intensity, expressed as the mean ± SEM (n = 10 independent DC preparations). (b) Cytokine levels in supernatants from DC cultures were measured by ELISA. (c) Each DC subset was incubated with FITC-dextran for 1 hr and the percentage of FITC-dextran-positive cells determined by flow cytometry. The bar graphs show the mean fluorescence intensity, expressed as the mean ± SEM (n = 5 independent DC preparations). (d) Each DC subset was co-cultured with CFSE-labeled CD3^+^ T cells to measure T cell proliferation. Each DC subset was co-cultured with CD3^+^ T cells (isolated from splenocytes obtained from naïve C57BL/6 mice) for 72 h. The stimulator:responder ratio was 1:10. (e) T cell subpopulations analyzed by flow cytometry. The percentages of induced Th1 (CD4^+^IFN-γ^+^), Th2 (CD4^+^IL-4^+^), and Th17 (CD4^+^IL-17A^+^) cells are shown on the plots. (f) Measurement of Th1/Th17 cytokines in culture supernatants from DCs/T cell co-cultures by ELISA. Data are expressed as mean ± SEM (n = 10 independent DC preparations). **P* < 0.05, ***P* < 0.01, and ****P* < 0.001, compared with imDC.
